# Supplementary material for: Multi‐dataset identification of innovative feature genes and molecular mechanisms in keratoconus
Source: J Cell Mol Med. 2024 Sep 19;28(18):e70079. doi: 10.1111/jcmm.70079 (PMC11412914; doi:10.1111/jcmm.70079)
Supplement: Supplementary file 2 — Material S2. [file JCMM-28-e70079-s002.zip › 7.GSEA analysis/6.SNORA74B/KEGG_PROTEASOME.html]

Details for gene set KEGG\_PROTEASOME[GSEA]

|  || Dataset | SNORA74B.group.cls#h\_versus\_l |
| Phenotype | group.cls#h\_versus\_l |
| Upregulated in class | h |
| GeneSet | KEGG\_PROTEASOME |
| Enrichment Score (ES) | 0.11619812 |
| Normalized Enrichment Score (NES) | 0.4084021 |
| Nominal p-value | 1.0 |
| FDR q-value | 0.9985415 |
| FWER p-Value | 1.0 |
Table: GSEA Results Summary

  

Fig 1: Enrichment plot: KEGG\_PROTEASOME      
 Profile of the Running ES Score & Positions of GeneSet Members on the Rank Ordered List

  

| SYMBOL | TITLE | RANK IN GENE LIST | RANK METRIC SCORE | RUNNING ES | CORE ENRICHMENT || 1 | PSMB11 | na | 2702 | 0.320 | 0.0556 | Yes |
| 2 | PSMD3 | na | 8639 | 0.122 | -0.0648 | Yes |
| 3 | PSMA4 | na | 9270 | 0.108 | -0.0381 | Yes |
| 4 | PSMB10 | na | 9796 | 0.097 | -0.0129 | Yes |
| 5 | PSMC2 | na | 10635 | 0.081 | -0.0033 | Yes |
| 6 | PSMB8 | na | 11127 | 0.073 | 0.0129 | Yes |
| 7 | PSMD13 | na | 11551 | 0.066 | 0.0283 | Yes |
| 8 | PSMA1 | na | 11904 | 0.061 | 0.0438 | Yes |
| 9 | PSMA8 | na | 12430 | 0.054 | 0.0513 | Yes |
| 10 | PSME4 | na | 12652 | 0.051 | 0.0663 | Yes |
| 11 | PSME1 | na | 13923 | 0.037 | 0.0450 | Yes |
| 12 | PSMB3 | na | 14052 | 0.035 | 0.0560 | Yes |
| 13 | PSMC5 | na | 14380 | 0.032 | 0.0597 | Yes |
| 14 | PSMB7 | na | 15544 | 0.019 | 0.0341 | Yes |
| 15 | PSMB4 | na | 15650 | 0.018 | 0.0386 | Yes |
| 16 | PSMD4 | na | 16411 | 0.010 | 0.0210 | Yes |
| 17 | PSMA3 | na | 16469 | 0.010 | 0.0235 | Yes |
| 18 | PSMA7 | na | 16569 | 0.009 | 0.0242 | Yes |
| 19 | PSMD8 | na | 17436 | 0.000 | -0.0007 | Yes |
| 20 | PSME2 | na | 17771 | -0.003 | -0.0089 | Yes |
| 21 | PSMD7 | na | 17801 | -0.004 | -0.0082 | Yes |
| 22 | PSMB1 | na | 18183 | -0.008 | -0.0158 | Yes |
| 23 | PSMA5 | na | 18538 | -0.012 | -0.0210 | Yes |
| 24 | PSMB5 | na | 18674 | -0.014 | -0.0192 | Yes |
| 25 | PSMB2 | na | 18680 | -0.014 | -0.0136 | Yes |
| 26 | PSMB9 | na | 19032 | -0.017 | -0.0165 | Yes |
| 27 | PSMA6 | na | 19479 | -0.023 | -0.0199 | Yes |
| 28 | PSMD1 | na | 19546 | -0.023 | -0.0120 | Yes |
| 29 | PSMD2 | na | 19611 | -0.024 | -0.0037 | Yes |
| 30 | PSMC1 | na | 19793 | -0.026 | 0.0020 | Yes |
| 31 | PSMD6 | na | 20148 | -0.030 | 0.0043 | Yes |
| 32 | PSMB6 | na | 20236 | -0.031 | 0.0146 | Yes |
| 33 | PSME3 | na | 20513 | -0.034 | 0.0210 | Yes |
| 34 | PSMD14 | na | 20739 | -0.037 | 0.0299 | Yes |
| 35 | PSMD11 | na | 20936 | -0.039 | 0.0406 | Yes |
| 36 | PSMA2 | na | 20937 | -0.039 | 0.0569 | Yes |
| 37 | PSMC3 | na | 21255 | -0.043 | 0.0657 | Yes |
| 38 | PSMC6 | na | 21592 | -0.047 | 0.0755 | Yes |
| 39 | PSMD12 | na | 22492 | -0.058 | 0.0738 | Yes |
| 40 | PSMC4 | na | 22840 | -0.063 | 0.0900 | Yes |
| 41 | PSMF1 | na | 22841 | -0.063 | 0.1162 | Yes |
| 42 | POMP | na | 27589 | -0.154 | 0.0435 | No |
| 43 | IFNG | na | 32943 | -0.389 | 0.0515 | No |
Table: GSEA details [plain text format]

  

Fig 2: KEGG\_PROTEASOME      
 Blue-Pink O' Gram in the Space of the Analyzed GeneSet

  

Fig 3: KEGG\_PROTEASOME: Random ES distribution      
 Gene set null distribution of ES for **KEGG\_PROTEASOME**
